# 9-Amino­acridinium nitrate monohydrate

**DOI:** 10.1107/S1600536811003953

**Published:** 2011-02-05

**Authors:** Mehrdad Pourayoubi, Hossein Eshtiagh-Hosseini, Somayyeh Sanaei Ataabadi, Teresa Mancilla Percino, Marco A. Leyva Ramírez

**Affiliations:** a Department of Chemistry, Ferdowsi University of Mashhad, Mashhad, 91779, Iran; b Department of Chemistry, Islamic Azad University, Shahr-e-Rey Branch, Tehran, Iran; c Departamento de Química, Centro de Investigación y de Estudios Avanzados del Instituto Politécnico Nacional, Apartado Postal 14-740, 07000 México, DF, Mexico

## Abstract

The pyridine N atom of the cation in the title hydrated salt, C_13_H_11_N_2_^+^·NO_3_^−^·H_2_O, is protonated; the N atom of the NH_2_ group shows a planar conformation. The former N atom is hydrogen bonded to a water mol­ecule. The amino group is involved in three N—H⋯O hydrogen bonds with two neighboring nitrate anions. The water mol­ecule is hydrogen bonded to two adjacent nitrate anions. In the crystal, this results in a layered network.

## Related literature

For the structure of 9-amino­acridine hydro­chloride monohydrate, see: Talacki *et al.* (1974 ▶). For positive-charge-assisted hydrogen bonds, see: Gilli *et al.* (1994 ▶). 
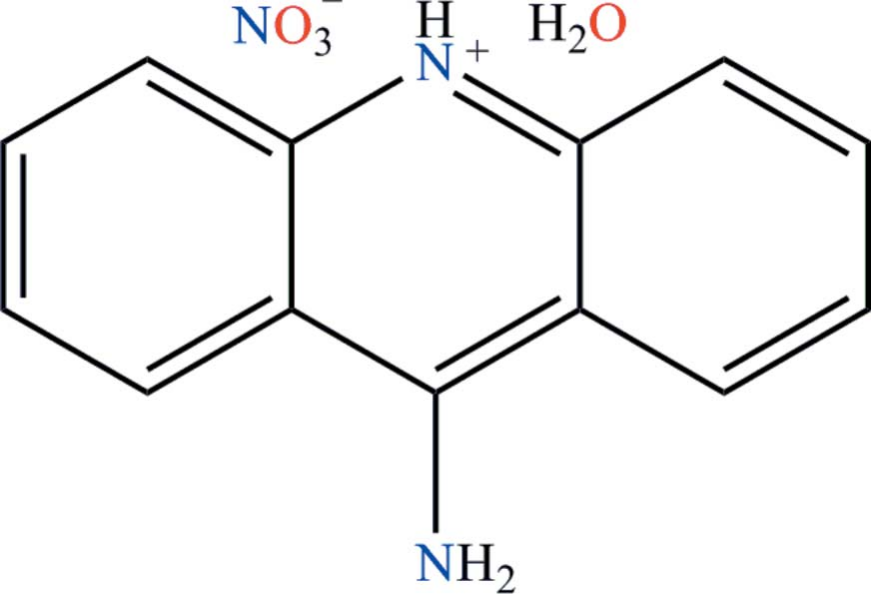


## Experimental

### 

#### Crystal data

C_13_H_11_N_2_^+^·NO_3_^−^·H_2_O
*M* *_r_* = 275.26
Triclinic, 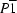

*a* = 6.8556 (2) Å
*b* = 10.0532 (2) Å
*c* = 10.5912 (3) Å
α = 117.016 (1)°
β = 94.138 (1)°
γ = 97.995 (1)°
*V* = 636.36 (3) Å^3^
*Z* = 2
Mo *K*α radiation
μ = 0.11 mm^−1^
*T* = 293 K
0.75 × 0.75 × 0.45 mm

#### Data collection

Nonius KappaCCD diffractometer
Absorption correction: multi-scan (Blessing, 1995 ▶) *T*_min_ = 0.923, *T*_max_ = 0.953
8945 measured reflections
2822 independent reflections
2054 reflections with *I* > 2σ(*I*)
*R*_int_ = 0.028

#### Refinement

*R*[*F* ^2^ > 2σ(*F* ^2^)] = 0.045
*wR*(*F* ^2^) = 0.132
*S* = 1.04
2822 reflections
201 parameters
5 restraints
H atoms treated by a mixture of independent and constrained refinement
Δρ_max_ = 0.26 e Å^−3^
Δρ_min_ = −0.22 e Å^−3^

### 

Data collection: *COLLECT* (Nonius, 2001 ▶); cell refinement: *SCALEPACK* (Otwinowski & Minor, 1997 ▶); data reduction: *DENZO* (Otwinowski & Minor, 1997 ▶) and *SCALEPACK*; program(s) used to solve structure: *SHELXS97* (Sheldrick, 2008 ▶); program(s) used to refine structure: *SHELXL97* (Sheldrick, 2008 ▶); molecular graphics: *Mercury* (Macrae *et al.*, 2006 ▶); software used to prepare material for publication: *WinGX* (Farrugia, 1999 ▶) and *enCIFer* (Allen *et al.*, 2004 ▶).

## Supplementary Material

Crystal structure: contains datablocks I, global. DOI: 10.1107/S1600536811003953/ng5102sup1.cif

Structure factors: contains datablocks I. DOI: 10.1107/S1600536811003953/ng5102Isup2.hkl

Additional supplementary materials:  crystallographic information; 3D view; checkCIF report

## Figures and Tables

**Table 1:** Hydrogen-bond geometry (Å, °)

*D*—H⋯*A*	*D*—H	H⋯*A*	*D*⋯*A*	*D*—H⋯*A*
N2—H2*A*⋯O1^i^	0.93 (1)	2.23 (2)	3.0619 (17)	149 (1)
N2—H2*A*⋯O3^i^	0.93 (1)	2.30 (2)	3.0662 (16)	140 (1)
N2—H2*B*⋯O2^ii^	0.90 (1)	2.07 (1)	2.9123 (15)	157 (2)
O4—H4*A*⋯O3^iii^	0.91 (2)	2.03 (2)	2.9147 (18)	164 (2)
N1—H1⋯O4	0.89 (1)	1.91 (1)	2.7867 (15)	170 (2)
O4—H4*B*⋯O1	0.90 (2)	2.01 (2)	2.9058 (18)	173 (2)
O4—H4*B*⋯O2	0.90 (2)	2.64 (2)	3.2039 (19)	122 (2)

Symmetry codes: (i) 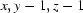; (ii) 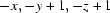; (iii) 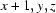.

**Fig. 1.**
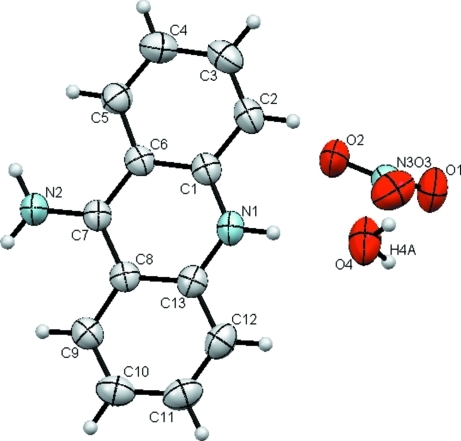
Molecular view with the atom labeling scheme, displacement ellipsoids are drawn at the 50% probability level. H atoms are represented as small spheres of arbitrary radii.

**Fig. 2.**
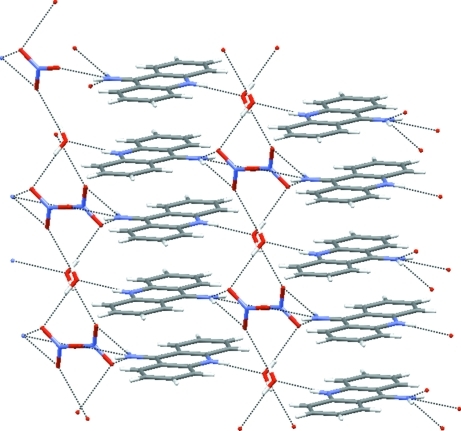
Partial packing of cations, anions and water molecules in the title hydrated salt. H bonds are shown as dashed lines.

## supplementary crystallographic information

### Comment

In a previous work, the crystal structure of 9-aminoacridine hydrochloride monohydrate (Talacki *et al.*, 1974) has been investigated. Here, we report on the crystal structure of title hydrated salt, C_13_H_11_N_2_^+^.NO_3_^-^.H_2_O (Fig. 1).

In 9-amino-acridinium cation, the heteroatom N1 and the nitrogen atom of NH_2_ unit (N2) have a *sp*^2^ character. The C1—N1—C13 angle is 122.68 (11)°; the fused tricyclic system is essentially planar.

The protonated pyridine nitrogen atom is involving in a positive charge assisted (Gilli *et al.*, 1994) N—H···O hydrogen bond with a neighboring H_2_O molecule (N1···O4 = 2.7867 (15) Å). Moreover, the water molecule forms two O—H···O hydrogen bonds (O···O = 2.9058 (18) & 2.9147 (18) Å) with two adjacent NO_3_^-^ anions; also, the weak hydrogen bond O4—H4B···O2 (O4···O2 = 3.2039 (19) Å) may be considered which has not influence on the pattern of crystal packing. The NH_2_ unit of cation cooperates in three N—H···O hydrogen bonds (N···O = 2.9123 (15), 3.0619 (17) and 3.0662 (16) Å), with two neighboring nitrate anions. Cations, anions and water molecules are hydrogen bonded in a 2-D arrangement (Fig. 2).

### Experimental

The title hydrated salt was obtained fortuitously from the reaction between 9-aminoacridine and Fe(NO_3_)_3_.9H_2_O in CH_3_OH as follows: To a solution of 9-aminoacridine (0.194 g, 1 mmol) in CH_3_OH (5 ml), a solution of Fe(NO_3_)_3_.9H_2_O (0.202 g, 0.5 mmol) in CH_3_OH (5 ml) was added at 343 K. After 1 h stirring, the solid was filtered; the crystals were obtained from methanolic solution after a slow evaporation at room temperature.

### Refinement

The hydrogen atom of NH group and those of water molecule were found in difference Fourier synthesis.The NH H atoms were restrained to 0.90 A and the refinement give good values. The H atoms in the water molecule were refined with a restraint of 1.00 A for a ideal distance OH and obtained acceptable values. The H(C) atom positions were calculated. All hydrogen atoms were refined in isotropic approximation in riding model with the *U*_iso_(H) parameters equal to 1.2 *U*_eq_(Ci), for methyl groups equal to 1.5 *U*_eq_(Cii), where U(Ci) and U(Cii) are respectively the equivalent thermal parameters of the carbon atoms to which corresponding H atoms are bonded.

### Crystal data

**Table:**

C_13_H_11_N_2_^+^·NO_3_^−^·H_2_O	*Z* = 2
*M**_r_* = 275.26	*F*(000) = 288
Triclinic, *P*1	*D*_x_ = 1.437 Mg m^−^^3^
Hall symbol: -P 1	Mo *K*α radiation, λ = 0.71073 Å
*a* = 6.8556 (2) Å	Cell parameters from 600 reflections
*b* = 10.0532 (2) Å	θ = 1–14°
*c* = 10.5912 (3) Å	µ = 0.11 mm^−^^1^
α = 117.016 (1)°	*T* = 293 K
β = 94.138 (1)°	Block, colourless
γ = 97.995 (1)°	0.75 × 0.75 × 0.45 mm
*V* = 636.36 (3) Å^3^	

### Data collection

**Table:**

Nonius KappaCCD diffractometer	2822 independent reflections
Radiation source: fine-focus sealed tube	2054 reflections with *I* > 2σ(*I*)
graphite	*R*_int_ = 0.028
CCD rotation images, thick slices scans	θ_max_ = 27.6°, θ_min_ = 3.0°
Absorption correction: multi-scan (Blessing, 1995)	*h* = −8→8
*T*_min_ = 0.923, *T*_max_ = 0.953	*k* = −12→13
8945 measured reflections	*l* = −13→13

### Refinement

**Table:**

Refinement on *F*^2^	Primary atom site location: structure-invariant direct methods
Least-squares matrix: full	Secondary atom site location: difference Fourier map
*R*[*F*^2^ > 2σ(*F*^2^)] = 0.045	Hydrogen site location: inferred from neighbouring sites
*wR*(*F*^2^) = 0.132	H atoms treated by a mixture of independent and constrained refinement
*S* = 1.04	*w* = 1/[σ^2^(*F*_o_^2^) + (0.0799*P*)^2^ + 0.0299*P*] where *P* = (*F*_o_^2^ + 2*F*_c_^2^)/3
2822 reflections	(Δ/σ)_max_ < 0.001
201 parameters	Δρ_max_ = 0.26 e Å^−^^3^
5 restraints	Δρ_min_ = −0.22 e Å^−^^3^

### Special details

**Table:**

Geometry. All s.u.'s (except the s.u. in the dihedral angle between two l.s. planes) are estimated using the full covariance matrix. The cell s.u.'s are taken into account individually in the estimation of s.u.'s in distances, angles and torsion angles; correlations between s.u.'s in cell parameters are only used when they are defined by crystal symmetry. An approximate (isotropic) treatment of cell s.u.'s is used for estimating s.u.'s involving l.s. planes.
Refinement. Refinement of *F*^2^ against ALL reflections. The weighted *R*-factor *wR* and goodness of fit *S* are based on *F*^2^, conventional *R*-factors *R* are based on *F*, with *F* set to zero for negative *F*^2^. The threshold expression of *F*^2^ > 2σ(*F*^2^) is used only for calculating *R*-factors(gt) *etc*. and is not relevant to the choice of reflections for refinement. *R*-factors based on *F*^2^ are statistically about twice as large as those based on *F*, and *R*- factors based on ALL data will be even larger.

### Fractional atomic coordinates and isotropic or equivalent isotropic displacement parameters (Å^2^)

**Table:**

	*x*	*y*	*z*	*U*_iso_*/*U*_eq_	
C1	0.28645 (17)	0.69390 (14)	0.48088 (14)	0.0392 (3)	
C2	0.3072 (2)	0.82982 (15)	0.46976 (16)	0.0491 (3)	
H2	0.3418	0.9237	0.5518	0.059*	
C3	0.2764 (2)	0.82263 (16)	0.33871 (17)	0.0548 (4)	
H3	0.2900	0.9123	0.3317	0.066*	
C4	0.2245 (2)	0.68227 (17)	0.21357 (17)	0.0545 (4)	
H4	0.2054	0.6794	0.1245	0.065*	
C5	0.20191 (19)	0.54989 (15)	0.22257 (14)	0.0456 (3)	
H5	0.1663	0.4572	0.1393	0.055*	
C6	0.23215 (17)	0.55232 (13)	0.35736 (13)	0.0377 (3)	
C7	0.20689 (17)	0.41608 (13)	0.37264 (13)	0.0368 (3)	
C8	0.23830 (16)	0.43023 (13)	0.51410 (13)	0.0369 (3)	
C9	0.21635 (19)	0.30332 (15)	0.54077 (15)	0.0438 (3)	
H9	0.1779	0.2057	0.4642	0.053*	
C10	0.2506 (2)	0.32195 (17)	0.67683 (16)	0.0513 (4)	
H10	0.2353	0.2375	0.6924	0.062*	
C11	0.3088 (2)	0.46790 (18)	0.79260 (16)	0.0554 (4)	
H11	0.3326	0.4797	0.8850	0.066*	
C12	0.3312 (2)	0.59339 (17)	0.77252 (14)	0.0512 (4)	
H12	0.3695	0.6900	0.8507	0.061*	
C13	0.29601 (17)	0.57631 (14)	0.63302 (13)	0.0393 (3)	
N1	0.31813 (16)	0.70224 (12)	0.61313 (12)	0.0437 (3)	
N2	0.15562 (19)	0.28113 (13)	0.25875 (12)	0.0507 (3)	
N3	0.0612 (2)	1.10579 (12)	0.88933 (12)	0.0521 (3)	
O1	0.22383 (17)	1.19337 (12)	0.95048 (12)	0.0721 (4)	
O2	0.04483 (18)	1.00530 (11)	0.76309 (11)	0.0674 (3)	
O3	−0.08334 (18)	1.12104 (15)	0.95548 (12)	0.0739 (4)	
O4	0.4979 (2)	0.98619 (13)	0.83821 (14)	0.0775 (4)	
H1	0.361 (2)	0.7928 (17)	0.6889 (16)	0.062 (5)*	
H2A	0.133 (2)	0.2695 (19)	0.1668 (16)	0.061 (4)*	
H2B	0.126 (2)	0.1971 (17)	0.2686 (18)	0.067 (5)*	
H4A	0.626 (2)	1.017 (2)	0.883 (2)	0.094 (7)*	
H4B	0.422 (3)	1.057 (2)	0.875 (2)	0.098 (7)*	

### Atomic displacement parameters (Å^2^)

**Table:**

	*U*^11^	*U*^22^	*U*^33^	*U*^12^	*U*^13^	*U*^23^
C1	0.0320 (6)	0.0385 (6)	0.0459 (7)	0.0080 (5)	0.0077 (5)	0.0183 (6)
C2	0.0462 (7)	0.0360 (6)	0.0613 (9)	0.0068 (5)	0.0078 (6)	0.0200 (6)
C3	0.0529 (8)	0.0489 (8)	0.0750 (10)	0.0128 (6)	0.0108 (7)	0.0386 (8)
C4	0.0580 (8)	0.0612 (9)	0.0577 (9)	0.0190 (7)	0.0121 (6)	0.0369 (8)
C5	0.0480 (7)	0.0453 (7)	0.0441 (7)	0.0138 (6)	0.0073 (5)	0.0202 (6)
C6	0.0321 (6)	0.0383 (6)	0.0432 (7)	0.0097 (5)	0.0083 (5)	0.0183 (6)
C7	0.0315 (6)	0.0356 (6)	0.0398 (7)	0.0079 (5)	0.0061 (5)	0.0142 (5)
C8	0.0288 (6)	0.0407 (7)	0.0418 (7)	0.0092 (5)	0.0076 (5)	0.0189 (6)
C9	0.0407 (7)	0.0435 (7)	0.0495 (7)	0.0097 (5)	0.0093 (5)	0.0229 (6)
C10	0.0475 (7)	0.0610 (9)	0.0605 (9)	0.0167 (6)	0.0154 (6)	0.0387 (8)
C11	0.0543 (8)	0.0750 (10)	0.0448 (8)	0.0185 (7)	0.0128 (6)	0.0325 (8)
C12	0.0494 (8)	0.0565 (8)	0.0390 (7)	0.0103 (6)	0.0078 (6)	0.0148 (6)
C13	0.0326 (6)	0.0428 (7)	0.0399 (7)	0.0084 (5)	0.0077 (5)	0.0166 (6)
N1	0.0450 (6)	0.0353 (6)	0.0412 (6)	0.0056 (5)	0.0052 (5)	0.0107 (5)
N2	0.0694 (8)	0.0356 (6)	0.0397 (6)	0.0073 (5)	0.0013 (5)	0.0133 (5)
N3	0.0707 (8)	0.0388 (6)	0.0428 (6)	0.0083 (6)	−0.0041 (6)	0.0182 (5)
O1	0.0757 (8)	0.0523 (6)	0.0587 (7)	−0.0043 (6)	−0.0034 (6)	0.0076 (5)
O2	0.0965 (8)	0.0445 (6)	0.0431 (6)	0.0017 (5)	−0.0006 (5)	0.0100 (5)
O3	0.0681 (7)	0.0950 (9)	0.0604 (7)	0.0244 (6)	0.0096 (6)	0.0358 (7)
O4	0.0701 (8)	0.0541 (7)	0.0758 (8)	0.0067 (6)	−0.0002 (6)	0.0060 (6)

### Geometric parameters (Å, °)

**Table:**

C1—N1	1.3641 (18)	C9—H9	0.9300
C1—C6	1.4024 (18)	C10—C11	1.396 (2)
C1—C2	1.4127 (18)	C10—H10	0.9300
C2—C3	1.356 (2)	C11—C12	1.362 (2)
C2—H2	0.9300	C11—H11	0.9300
C3—C4	1.403 (2)	C12—C13	1.4061 (19)
C3—H3	0.9300	C12—H12	0.9300
C4—C5	1.3654 (19)	C13—N1	1.3650 (17)
C4—H4	0.9300	N1—H1	0.887 (14)
C5—C6	1.4163 (18)	N2—H2A	0.925 (14)
C5—H5	0.9300	N2—H2B	0.895 (14)
C6—C7	1.4393 (17)	N3—O2	1.2411 (15)
C7—N2	1.3186 (16)	N3—O1	1.2417 (16)
C7—C8	1.4361 (17)	N3—O3	1.2417 (17)
C8—C13	1.4091 (18)	O4—H4A	0.909 (16)
C8—C9	1.4178 (17)	O4—H4B	0.901 (16)
C9—C10	1.364 (2)		
			
N1—C1—C6	120.53 (11)	C10—C9—H9	119.4
N1—C1—C2	119.19 (12)	C8—C9—H9	119.4
C6—C1—C2	120.28 (12)	C9—C10—C11	119.94 (13)
C3—C2—C1	119.60 (13)	C9—C10—H10	120.0
C3—C2—H2	120.2	C11—C10—H10	120.0
C1—C2—H2	120.2	C12—C11—C10	121.11 (13)
C2—C3—C4	121.12 (13)	C12—C11—H11	119.4
C2—C3—H3	119.4	C10—C11—H11	119.4
C4—C3—H3	119.4	C11—C12—C13	119.71 (13)
C5—C4—C3	120.01 (13)	C11—C12—H12	120.1
C5—C4—H4	120.0	C13—C12—H12	120.1
C3—C4—H4	120.0	N1—C13—C12	119.63 (12)
C4—C5—C6	120.69 (13)	N1—C13—C8	120.00 (11)
C4—C5—H5	119.7	C12—C13—C8	120.37 (12)
C6—C5—H5	119.7	C1—N1—C13	122.68 (11)
C1—C6—C5	118.30 (11)	C1—N1—H1	118.7 (11)
C1—C6—C7	118.91 (11)	C13—N1—H1	118.6 (11)
C5—C6—C7	122.78 (11)	C7—N2—H2A	122.2 (10)
N2—C7—C8	120.83 (11)	C7—N2—H2B	120.4 (11)
N2—C7—C6	120.48 (11)	H2A—N2—H2B	116.9 (16)
C8—C7—C6	118.70 (11)	O2—N3—O1	119.79 (14)
C13—C8—C9	117.72 (11)	O2—N3—O3	120.94 (13)
C13—C8—C7	119.16 (11)	O1—N3—O3	119.27 (12)
C9—C8—C7	123.11 (11)	H4A—O4—H4B	114 (2)
C10—C9—C8	121.15 (13)		
			
N1—C1—C2—C3	179.86 (12)	N2—C7—C8—C9	−0.21 (19)
C6—C1—C2—C3	−0.71 (19)	C6—C7—C8—C9	179.70 (10)
C1—C2—C3—C4	−0.1 (2)	C13—C8—C9—C10	−0.24 (18)
C2—C3—C4—C5	0.7 (2)	C7—C8—C9—C10	179.00 (11)
C3—C4—C5—C6	−0.6 (2)	C8—C9—C10—C11	−0.1 (2)
N1—C1—C6—C5	−179.71 (11)	C9—C10—C11—C12	0.4 (2)
C2—C1—C6—C5	0.87 (17)	C10—C11—C12—C13	−0.2 (2)
N1—C1—C6—C7	1.12 (17)	C11—C12—C13—N1	179.87 (12)
C2—C1—C6—C7	−178.31 (10)	C11—C12—C13—C8	−0.1 (2)
C4—C5—C6—C1	−0.23 (19)	C9—C8—C13—N1	−179.64 (10)
C4—C5—C6—C7	178.91 (11)	C7—C8—C13—N1	1.09 (17)
C1—C6—C7—N2	179.90 (11)	C9—C8—C13—C12	0.38 (17)
C5—C6—C7—N2	0.77 (19)	C7—C8—C13—C12	−178.89 (11)
C1—C6—C7—C8	−0.01 (16)	C6—C1—N1—C13	−1.16 (18)
C5—C6—C7—C8	−179.14 (11)	C2—C1—N1—C13	178.27 (10)
N2—C7—C8—C13	179.02 (11)	C12—C13—N1—C1	−179.99 (11)
C6—C7—C8—C13	−1.08 (16)	C8—C13—N1—C1	0.03 (18)

### Hydrogen-bond geometry (Å, °)

**Table:**

*D*—H···*A*	*D*—H	H···*A*	*D*···*A*	*D*—H···*A*
N2—H2A···O1^i^	0.93 (1)	2.23 (2)	3.0619 (17)	149.(1)
N2—H2A···O3^i^	0.93 (1)	2.30 (2)	3.0662 (16)	140.(1)
N2—H2B···O2^ii^	0.90 (1)	2.07 (1)	2.9123 (15)	157.(2)
O4—H4A···O3^iii^	0.91 (2)	2.03 (2)	2.9147 (18)	164.(2)
N1—H1···O4	0.89 (1)	1.91 (1)	2.7867 (15)	170.(2)
O4—H4B···O1	0.90 (2)	2.01 (2)	2.9058 (18)	173 (2)
O4—H4B···O2	0.90 (2)	2.64 (2)	3.2039 (19)	122.(2)

Symmetry codes: (i) *x*, *y*−1, *z*−1; (ii) −*x*, −*y*+1, −*z*+1; (iii) *x*+1, *y*, *z*.
